# The Anti-Proliferative Activity of BTG/TOB Proteins Is Mediated via the Caf1a (CNOT7) and Caf1b (CNOT8) Deadenylase Subunits of the Ccr4-Not Complex

**DOI:** 10.1371/journal.pone.0051331

**Published:** 2012-12-07

**Authors:** Rachel Doidge, Saloni Mittal, Akhmed Aslam, G. Sebastiaan Winkler

**Affiliations:** School of Pharmacy, Centre for Biomolecular Sciences, University of Nottingham, University Park, Nottingham, United Kingdom; Ecole Normale Superieure de Lyon, France

## Abstract

The human BTG/TOB protein family comprises six members (BTG1, BTG2/PC3/Tis21, BTG3/Ana, BTG4/PC3B, TOB1/Tob, and TOB2) that are characterised by a conserved BTG domain. This domain mediates interactions with the highly similar Caf1a (CNOT7) and Caf1b (CNOT8) catalytic subunits of the Ccr4-Not deadenylase complex. BTG/TOB proteins have anti-proliferative activity: knockdown of BTG/TOB can result in increased cell proliferation, whereas over-expression of BTG/TOB leads to inhibition of cell cycle progression. It was unclear whether the interaction between BTG/TOB proteins and the Caf1a/Caf1b deadenylases is necessary for the anti-proliferative activity of BTG/TOB. To address this question, we further characterised surface-exposed amino acid residues of BTG2 and TOB1 that mediate the interaction with the Caf1a and Caf1b deadenylase enzymes. We then analysed the role of BTG2 and TOB1 in the regulation of cell proliferation, translation and mRNA abundance using a mutant that is no longer able to interact with the Caf1a/Caf1b deadenylases. We conclude that the anti-proliferative activity of BTG/TOB proteins is mediated through interactions with the Caf1a and Caf1b deadenylase enzymes. Furthermore, we show that the activity of BTG/TOB proteins in the regulation of mRNA abundance and translation is dependent on Caf1a/Caf1b, and does not appear to require other Ccr4-Not components, including the Ccr4a (CNOT6)/Ccr4b (CNOT6L) deadenylases, or the non-catalytic subunits CNOT1 or CNOT3.

## Introduction

The BTG/TOB family of anti-proliferative proteins is composed of six members in mammalian cells (BTG1, BTG2/PC3/Tis21, BTG3/Ana, BTG4/PC3B, TOB/TOB1, and TOB2) and are characterised by the conserved N-terminal BTG domain spanning 104–106 amino acids [1], [2]. The TOB1 and TOB2 proteins have a large additional C-terminal region, which contains PAM2 motifs that mediate interactions with poly(A)-binding proteins [3], [4], [5].

Expression of BTG/TOB proteins results in the inhibition of cell cycle progression [1], [2], [6], [7]. In agreement with their anti-proliferative activity, loss of expression of BTG2, BTG3, and TOB1 is frequently observed in clinical samples of various cancers, including lung, thyroid and breast tumours [8], [9], [10], [11]. Supporting a causal relationship between loss of expression and disease, gene inactivation of BTG3 and TOB1 is associated with predisposition to cancer in mouse models [10], [12]. In addition, whole-exome sequencing revealed that mutations in the BTG1 and BTG2 genes are frequently found in diffuse large B-cell lymphoma [13], [14]. Moreover, small deletions in BTG1 due to inappropriate RAG-mediated recombination are associated with acute lymphoblastic leukaemia [15]. At the molecular level, TOB1 inactivation via phosphorylation by MAPK signalling components has been implicated in Ras-mediated transformation [16]. Furthermore, BTG2 was identified as a key effector of p53-mediated inhibition of transformation of mouse embryonic fibroblasts [17]. Together, these findings highlight an emerging role for the BTG/TOB proteins as important tumour suppressors. In addition to a role in protecting cells from oncogenic transformation, members of the BTG/TOB family are also implicated in other physiological and cellular processes, such as the regulation of osteoblast activity, T-cell quiescence, and the G_2_/M DNA damage checkpoint [18], [19], [20].

The BTG domain can bind to the Caf1a and Caf1b enzymatic subunits of the Ccr4-Not complex, a multi-subunit deadenylase complex [21], [22], [23], [24], [25]. This complex is one of the major deadenylases in yeast, Drosophila and human cells and contains two types of catalytic subunits: a Caf1-type, which belongs to the DEDD/RNAse H superfamily, and a Ccr4-type, which contains an Endonuclease-Exonuclease-Phosphatase (EEP) domain [26], [27], [28], [29]. In human cells, the CNOT7 and CNOT8 genes encode the two paralogous deadenylases Caf1a and Caf1b, respectively, which have overlapping functions in the regulation of mRNA abundance [30]. Similarly, CNOT6 and CNOT6L encode the Ccr4a and Ccr4b paralogues, respectively, which are important for cell growth [31], [32]. Importantly, while Caf1a/Caf1b directly interact with the Ccr4a/Ccr4b enzymes, these deadenylases have a largely distinct role in the regulation of mRNA abundance [31]. Consistent with the reported interactions with deadenylase enzymes, it was shown that BTG2 and TOB1 contribute to mRNA deadenylation and decay [3], [33], [34]. In agreement with a recruitment model, whereby the Ccr4-Not complex is recruited via TOB1 interactions with the translational termination factor eRF3 or the RNA binding protein CPEB3, artificial tethering of TOB1 results in enhancement of mRNA deadenylation and decay [34], [35], [36].

An important unresolved question is whether the anti-proliferative activity of BTG/TOB proteins is related to their ability to interact with the Caf1a and Caf1b deadenylase subunits of the Ccr4-Not complex. Different conclusions were obtained using mutations in Caf1a that prevent the interaction of the deadenylase with BTG/TOB proteins [30], [37]. Furthermore, it is unclear whether other Ccr4-Not components, such as the Ccr4a and Ccr4b deadenylases, or the non-catalytic subunits CNOT1 and CNOT3, are required for the activity of BTG/TOB proteins in the regulation of mRNA deadenylation, decay and translation.

We addressed these questions using MCF-7 cells derived from an estrogen receptor-positive breast carcinoma. After establishing which BTG/TOB proteins were the most relevant family members to study in more detail, we further characterised the interaction surface of Caf1a/Caf1b with the BTG2 and TOB1 proteins. Using the substitution of a conserved single amino acid essential for the interaction between BTG2/TOB1 and Caf1a/Caf1b, we established the importance of the interaction between BTG2 and TOB1 with the deadenylase enzymes for their anti-proliferative activity. Finally, we used RNA tethering assays to demonstrate that recruitment of BTG2 or TOB1 to the 3′ UTR of mRNA affects translation and mRNA abundance, which is dependent on the interactions with the Caf1a/Caf1b deadenylases, but not on the presence of the Ccr4a/Ccr4b deadenylase enzymes, or the CNOT1 or CNOT3 non-catalytic subunits of the Ccr4-Not complex.

## Materials and Methods

### Plasmid DNA, siRNA and Antibody Reagents

The cDNAs encompassing the coding regions of BTG2 and TOB1 (flanked by XhoI sites) were obtained using reverse transcriptase PCR using total RNA isolated from MCF-7 cells and cloned into the EcoRV site of pBluescript II KS(+). The BTG2 cDNA was sub-cloned (BamHI-EcoRV) into pcDNA3-CMV-Flag. The Flag-BTG2 cDNA was then transferred (HindIII-EcoRV) into pcDNA4-TO to create pcDNA4-TO-Flag-BTG2. Subsequently, the BTG2 cDNAs was removed and replaced with an HA-BTG2 cDNA fragment (BamHI-EcoRV), which was generated by PCR to create plasmid pcDNA4-TO-Flag-HA-BTG2. Plasmids pCMV5-HA-BTG2 and pCMV5-HA-TOB1 were generated by ligation of XhoI cDNA fragments into SalI-digested vector. Expression plasmids containing HA and Flag-tagged Caf1a or Caf1b were described before [30].

For yeast two-hybrid analysis, the CNOT7 and CNOT8 cDNAs were inserted into the XhoI site of pGAL4-AD-2.1 (Stratagene). BTG2 and TOB1 cDNAs were inserted as EcoRI-XhoI fragments into the EcoRI and SalI sites of pGAL4-BD-HA, which contained an HA tag sequence inserted into the EcoRI and SalI sites of the vector pGAL4-BD Cam (Stratagene).

The reporter plasmid pRL-5BoxB was used for the tethering assay [38]. To construct the λN expression plasmids, we used PCR to generate cDNAs encoding HA-BTG2 and HA-TOB1, which were inserted as XhoI and XhoI-XbaI fragments, respectively, into plasmid pCI-λN [39], [40].

Mutations in the BTG2 and TOB1 cDNAs were introduced using a modified Quikchange procedure (Stratagene). The cDNA sequences present in all plasmids were confirmed by DNA sequencing.

Knockdown of CNOT7, CNOT8, CNOT6, CNOT6L, CNOT1, and CNOT3 was carried out using siGenome duplexes as described before [30], [31]. Knockdown of BTG/TOB components was performed using the following ON-TARGETplus siRNA pools (Thermo Fischer): L-011598-00 (BTG1), L-012308-00 (BTG2), L-010560-00 (TOB1), L-019434-00 (TOB2), and D-001810-10 (non-targeting control pool).

Antibodies used were: rat monoclonal 3F10 (HA epitope tag, Roche), mouse monoclonals M2 (Flag epitope tag, Sigma F1805), AC-15 (β-actin, Sigma A5441), 4B8 (CNOT3, Abnova), C-20 (γ-tubulin, Santa Cruz sc-7396), and C-10 (GAL4 activation domain, Santa Cruz sc-1663), and mouse polyclonal antibodies recognising CNOT1 (Proteintech, 14276-1-AP) and Caf1a (CNOT7)/Caf1b (CNOT8) (custom-made). All antibodies were used at a dilution of 1∶1000 for western blotting. Secondary antibodies were obtained from Santa Cruz.

### Yeast Two-hybrid Analysis

Yeast strain YRG-2 was transformed with pGAL4-AD 2.1 and pGAL4-BD Cam plasmids containing the indicated cDNAs using standard LiAc protocols (Stratagene). β-Galactosidase activity was determined using the Beta-GLO assay system (Promega) and normalised by measuring the optical density (600 nm) of the yeast cultures. Expression of Gal4DB-HA-BTG2 and Gal4AD-Caf1a/Gal4AD-Caf1b was confirmed by western blotting using antibodies recognising HA and Gal4-AD.

### Immunoprecipitation

HEK293 cells were transfected in 60 mm cell culture dishes with plasmids pcDNA4-TO-Flag-BTG2 and pCMV-HA-Caf1a (2.5 µg each) or pCMV5-HA-BTG2 and pCMV-Flag-Caf1a (2.5 µg each) using the jetPEI transfection reagent as per manufacturer’s instructions (Polyplus-transfection). Cells were lysed and immunoprecipitations using anti-Flag antibodies carried out as described before [30]. Bound proteins were eluted in SDS-PAGE loading buffer by incubation at 95°C for 4 min, and analysed by 12% SDS-PAGE and western blotting.

### Generation of Stable Cell Lines with Conditional Expression of BTG2

MCF-7 TR cells were generated by transfection of MCF-7 cells with plasmid pcDNA6/TR that expresses the Tet repressor protein (Invitrogen). Stably expressing clones were selected with blasticidin (5 µg/ml). Stable cell lines with doxycycline-dependent expression of BTG2 and BTG2 W103A were generated by transfection of plasmid pcDNA4-TO containing a Flag-HA-BTG2 cDNA. Clonal populations were selected using zeocin (300 µg/ml) and analysis of BTG2 protein levels by western blotting using HA antibodies.

### Analysis of Cell Proliferation

Stable MCF-7 cell lines with doxycycline-dependent expression of BTG2, BTG2 W103A cells, or empty vector-transfected cells were plated (n = 10,000 per well) in the presence or absence of doxycycline (1.0 µg/ml) in 6-well plates. After 3 days, cell numbers were counted at 24 h intervals using a haemocytometer for a period of five days.

**Figure 1:**
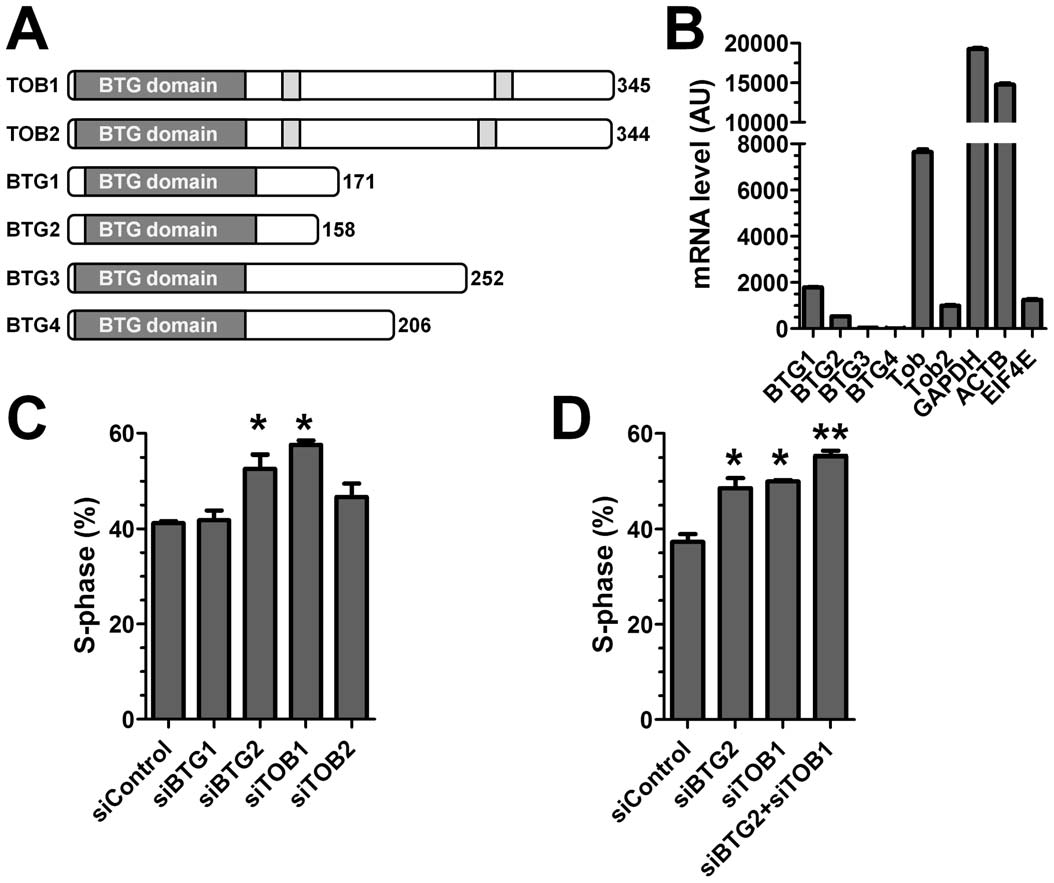
Knockdown of BTG2 and TOB1 results in increased proliferation of MCF-7 cells. (A) Overview of the human BTG/TOB family. The BTG domain is highlighted in grey, and PAM2 motifs present in TOB1 and TOB2 are highlighted in light grey. (B) Expression of BTG/TOB family members in MCF-7 cells. Data was obtained by genome-wide expression data using microarrays and confirmed by RT-qPCR. (C) Knockdown of BTG2 and TOB1 results in increased proliferation of MCF7 cells. Cells in S-phase were detected by labelling using the nucleoside analogue EdU 48 h after siRNA transfection. No effect was observed upon knockdown of BTG1 and TOB2. * p<0.05 (compared to treatment with non-targeting control siRNA). (D) Combined knockdown of BTG2 and TOB1 results in further increased cell proliferation of MCF7 cells. Cells in S-phase were detected by labelling using the nucleoside analogue EdU 48 h after siRNA transfection. * p<0.05 (compared to non-targeting control siRNA). ** p<0.05 (compared to BTG2 or TOB1 knockdown). All experiments were carried out in triplicate. Error bars represent the standard error of the mean.

### Immunofluorescence Detection of Cells in S-phase

MCF-7 cells (n = 160,000 per well) were plated in triplicates onto coverslips placed in 6-well plates and transfected with 1.0 µg pCMV5-HA-BTG2, pCMV5-HA-TOB1 or empty vector using the Genejuice transfection reagent as per manufacturer’s instructions (Merck Millipore). After pulse labelling for 2 h with 20 µM EdU, a thymidine analogue (invitrogen), cells were fixed and permeabilised 48 h post transfection and labelled using the Click-iT reaction as per manufacturer’s description (Invitrogen). After staining nuclei with Hoechst reagent (5 µg/ml), the percentage of cells in S-phase was determined by counting EdU stained cells as a percentage of Hoechst stained cells (n>100) using fluorescence microscopy.

### RNA Tethering Assays

MCF-7 cells (n = 180,000 per well) were plated in triplicate in 6-well plates and transfected with plasmids pRL-5BoxB and pCI-λN BTG2 or pCI-λN TOB1 (2.5 µg each) using jetPEI (Polyplus-transfection) [38], [39], [40]. Renilla luciferase activity was determined 24 h post transfection using the Gaussia luciferase kit following the manufacturer’s instructions (NEB) and normalised to total protein content. To measure the level of the reporter mRNA, total RNA was isolated using the RNeasy Plus kit (Qiagen) and RT-qPCR was carried out as described before with primers RT-hRL FW (ATGGGTAAGTCCGGCAAGA) and RT-hRL RV (CCAAGCGGTGAGGTACTTGT) [30], [31]. GAPDH was used as a reference gene.

For tethering assays combined with siRNA knockdown, MCF-7 cells were first transfected with a total of 10 nM siRNA duplexes using Interferin as described before [31]. The subsequent RNA tethering assay was carried out by transfecting the required plasmids 48 h after the siRNA transfection as described above.

**Figure 2:**
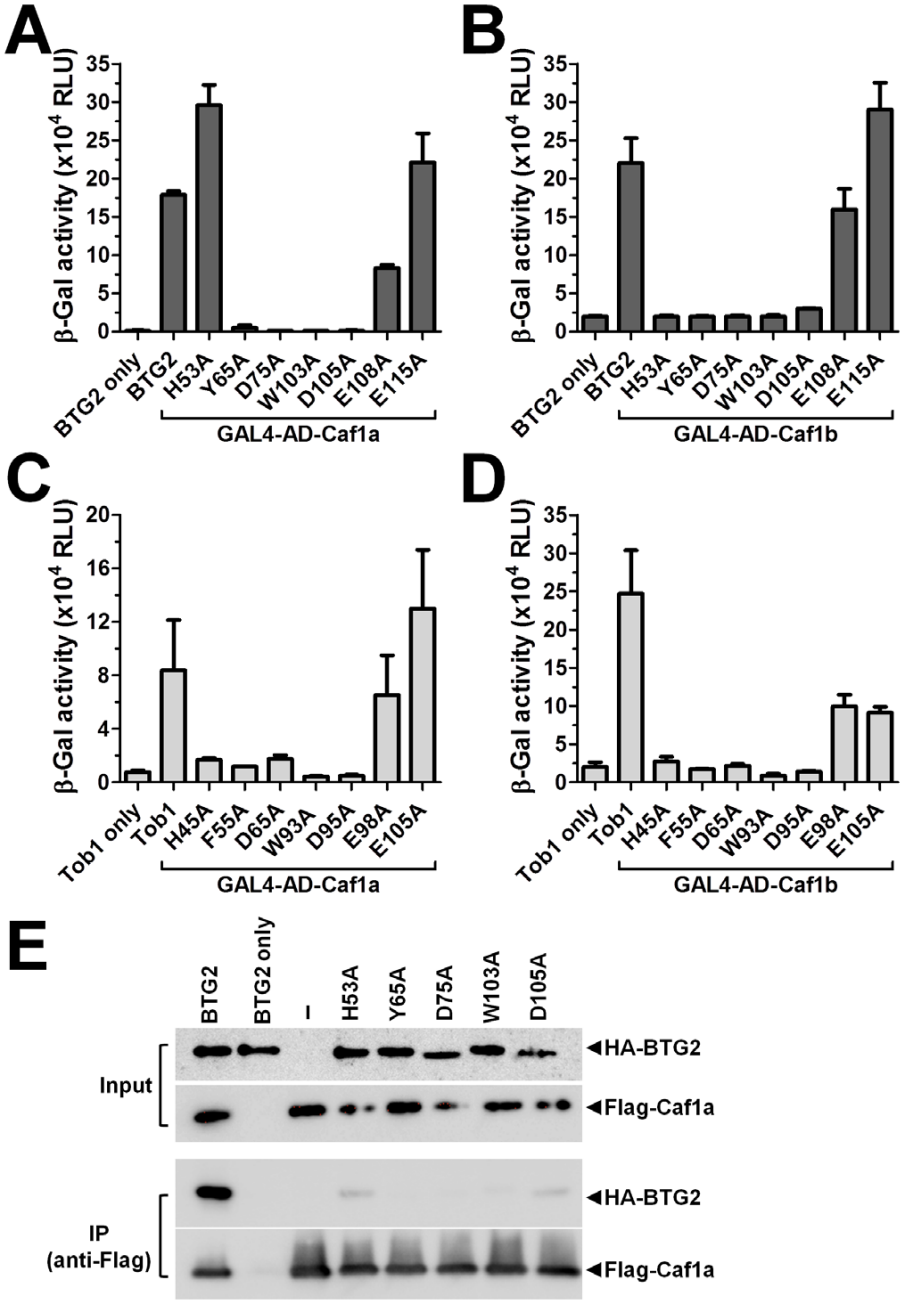
Amino acid residues of BTG2 and TOB1 mediating interactions with the Caf1a and Caf1b deadenylase enzymes. (A) Two-hybrid interaction analysis of BTG2 containing amino acid substitutions fused to the GAL4 DNA-binding domain and Caf1a fused to the activation domain of GAL4. (B) Two-hybrid interaction analysis of BTG2 containing amino acid substitutions fused to the GAL4 DNA-binding domain and Caf1b fused to the activation domain of GAL4. Plasmids encoding Gal4-DNA binding domain-BTG2 and Gal4-activation domain-Caf1a/Caf1b were transformed into yeast strain YRG-2 containing a LacZ reporter gene under control of five GAL4 consensus sequences. All experiments were carried out in triplicate. Error bars represent the standard error of the mean. (C) Two-hybrid interaction analysis of TOB1 containing amino acid substitutions fused to the GAL4 DNA-binding domain and Caf1a fused to the activation domain of GAL4. (D) Two-hybrid interaction analysis of TOB1 containing amino acid substitutions fused to the GAL4 DNA-binding domain and Caf1b fused to the activation domain of GAL4. Plasmids encoding Gal4-DNA binding domain-TOB1 and Gal4-activation domain-Caf1a/Caf1b were transformed into yeast strain YRG-2 containing a LacZ reporter gene under control of five GAL4 consensus sequences. All experiments were carried out in triplicate. Error bars represent the standard error of the mean. (E) Interaction analysis of HA-BTG2 and Flag-Caf1a using co-immunoprecipitations. Expression plasmids encoding Flag-Caf1a and HA-BTG2 were transfected into HEK293 cells. Following immunoprecipitation using anti-Flag antibodies, the presence of Flag-Caf1a and HA-BTG2 was assayed using western blotting using anti-Flag and anti-HA antibodies, respectively.

**Figure 3:**
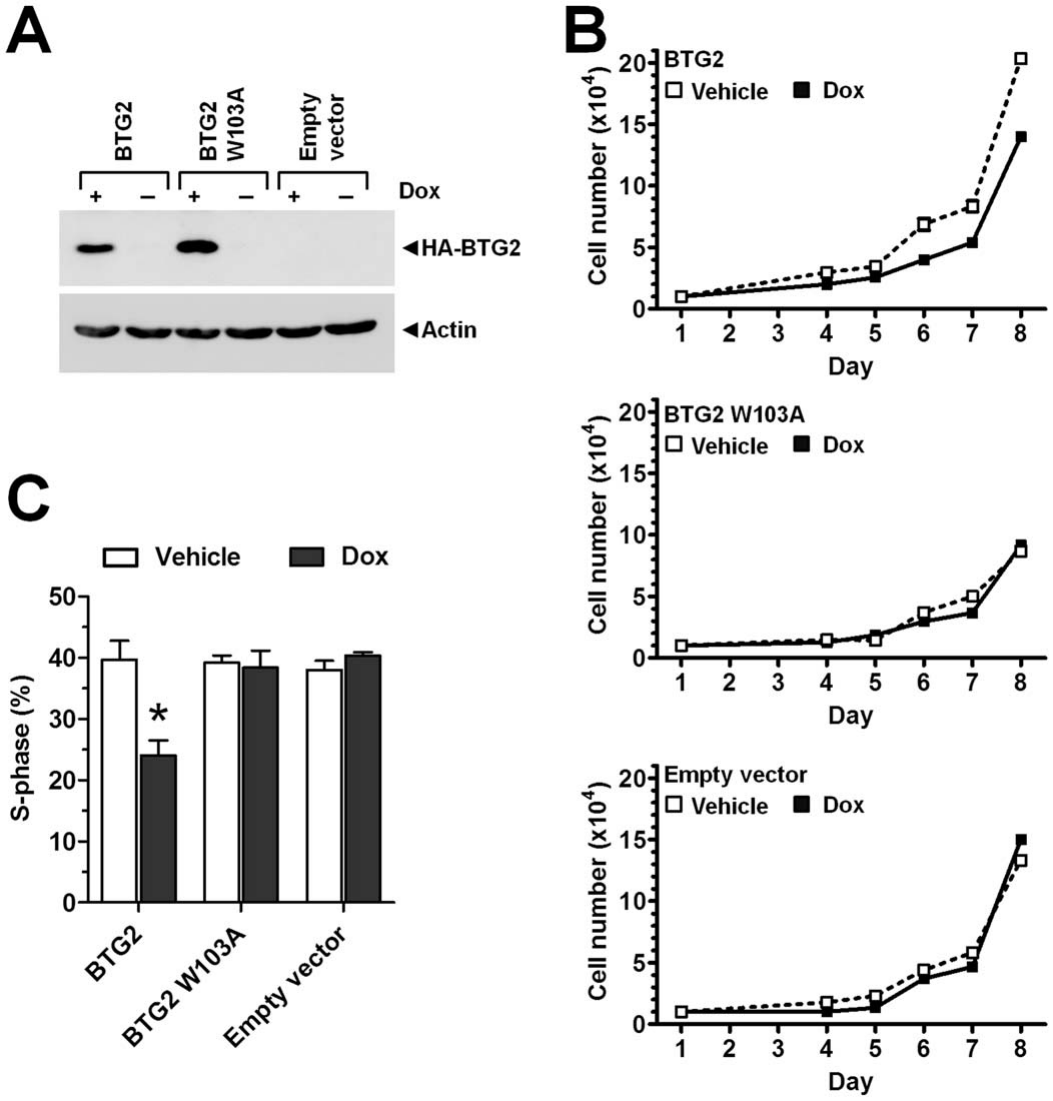
The interaction with the Caf1a/Caf1b deadenylase enzymes is required for the anti-proliferative activity of BTG2. (A) Inducible expression of BTG2 and BTG2 W103A in MCF-7 cells. Stable MCF-7 cell lines expressing a Tet repressor protein and containing an expression cassette containing HA-BTG2 or HA-BTG2 W103A under control of Tet repressor consensus sequences were generated. Expression of BTG2 and BTG2 W103A was induced by the addition of doxycycline (Dox) to the culture medium. (B) Reduced proliferation upon expression of BTG2, which is not observed upon expression of BTG2 W103A or in empty-vector transfected cells. Cell lines with inducible expression of BTG2 or BTG2 W103A and control cell lines transfected with empty vector were plated (n = 10 000 per well) and cultured in the presence of complete medium containing Doxycylin (Dox) or vehicle. Cell numbers were counted at the indicated intervals using a haemocytometer. All experiments were carried out in triplicate. Error bars represent the standard error of the mean are not visible due to the size of the markers. (C) Reduced number of cells in S-phase upon expression of BTG2, which is not observed upon expression of BTG2 W103A. Cell lines with inducible expression of BTG2 or BTG2 W103A and control cell lines transfected with empty vector were pulse-labelled for 2 h with the nucleoside analogue EdU 48 h after the addition of Doxycylin (Dox) or vehicle to the culture medium. EdU positive cells were detected with fluorescence microscopy. * p<0.05. All experiments were carried out in triplicate. Error bars represent the standard error of the mean.

**Figure 4:**
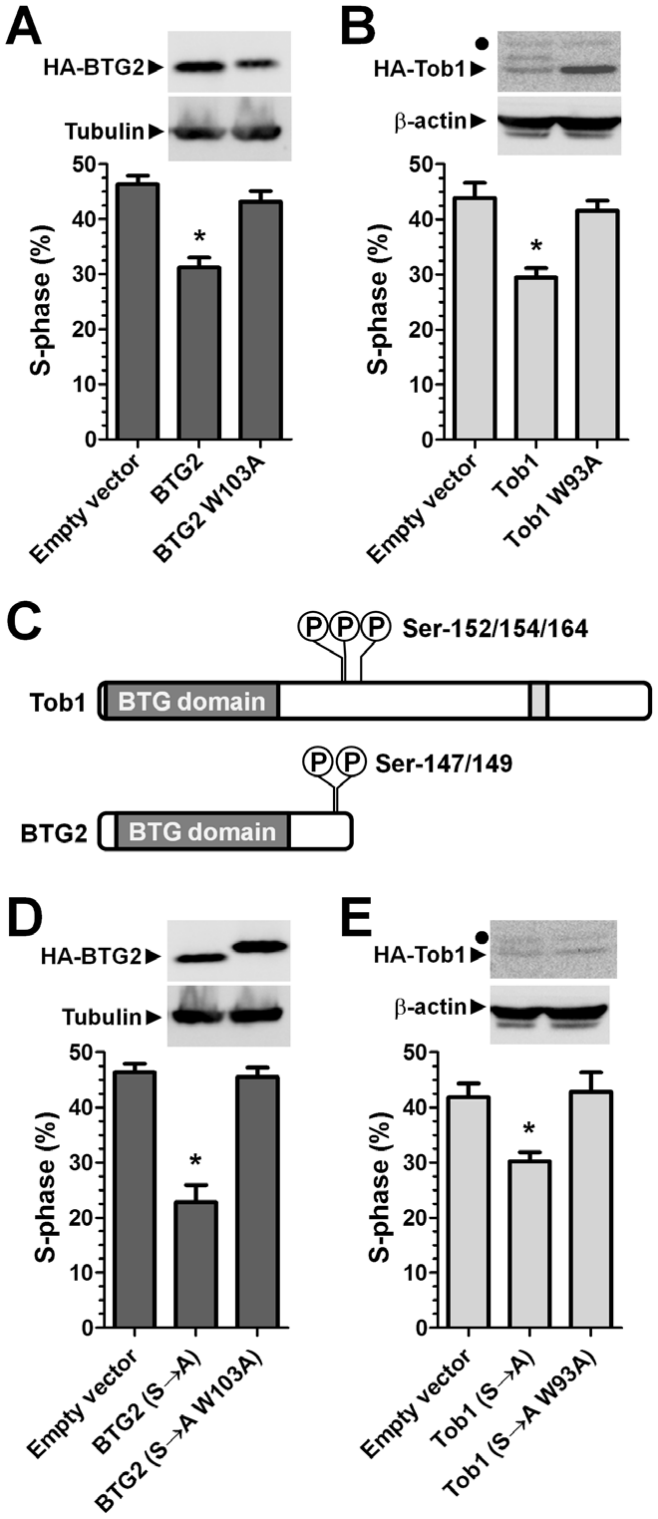
The anti-proliferative activity of BTG2 and TOB1 requires interactions with Caf1a/Caf1b. (A) Over-expression of BTG2, but not of BTG2 W103A, inhibits cell proliferation. 48 h after DNA transfection, MCF-7 cells were pulse-labelled (2 h) with the nucleoside analogue EdU. Cells in S-phase were detected by fluorescence microscopy. (B) Over-expression of TOB1, but not of TOB1 W93A, results in reduced cell proliferation. A filled circle in the top panel indicates a cross-reactive band. (C) Schematic diagram of TOB1 and BTG2 indicating the position of phosphorylation sites of TOB1 (Ser-152, Ser-154, Ser-164) and BTG2 (Ser-147 and Ser-149), which were altered to alanine (S→A). (D) Over-expression of BTG2 (S→A), but not of BTG2 W103A (S→A), decreases cell proliferation. (E) Over-expression of TOB1 (S→A), but not of TOB1 W93A (S→A), inhibits cell proliferation. A filled circle in the top panel indicates a cross-reactive band. Cells (n = 180,000) were plated in 6-well plates. After DNA transfection, cells were prepared for labelling and detection after 48 h. * p<0.05. All experiments were carried out in triplicate. Error bars represent the standard error of the mean.

**Figure 5:**
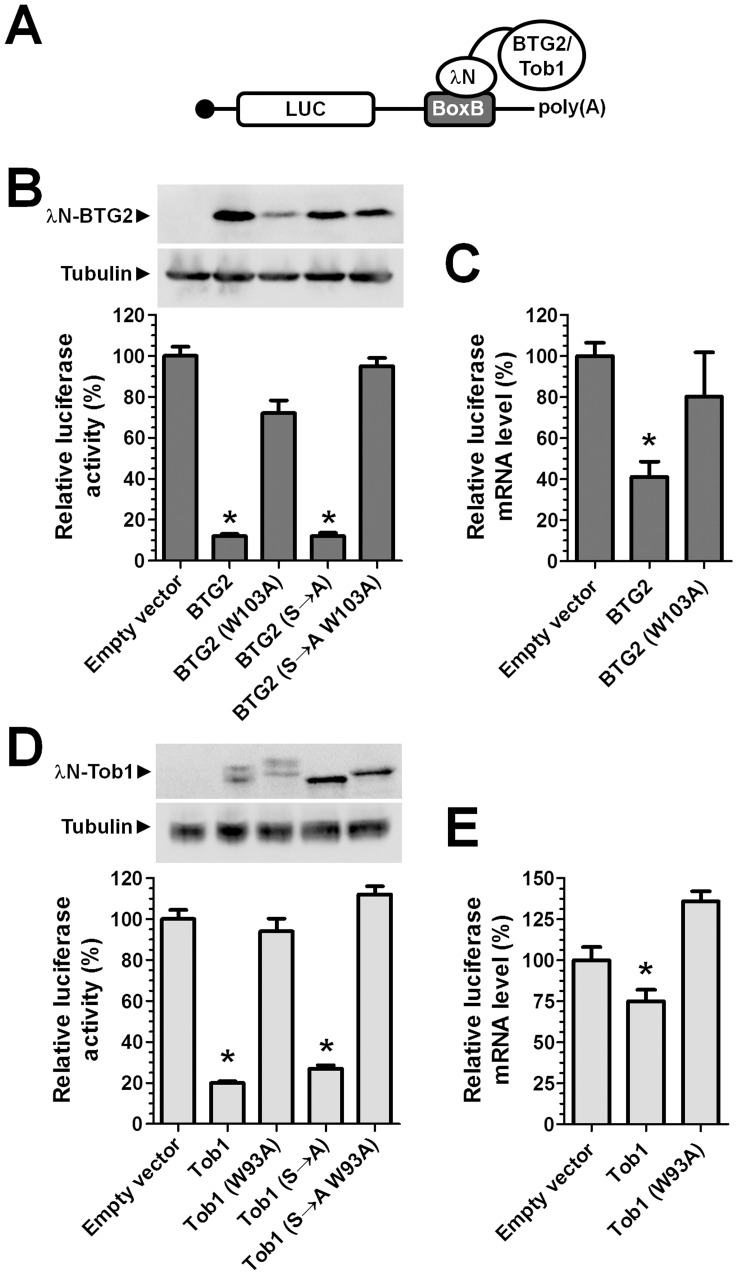
Recruitment of BTG2 or TOB1 to the 3′ UTR of mRNA results in reduced mRNA abundance and translation. (A) Schematic representation of the reporter mRNA containing coding sequences for the Renilla luciferase enzyme and five BoxB sequences in the 3′ UTR. Recruitment of BTG2 or TOB1 to the 3′ UTR was mediated by the fused λN peptide, which has high affinity for the BoxB sequence. (B) Recruitment of BTG2, but not of BTG2 W103A, to the 3′ UTR of a reporter gene inhibits reporter activity. MCF-7 cells were transfected with the indicated expression plasmids. Luciferase activity was determined 48 h post transfection and normalised to total protein content. Protein levels of λN-BTG2 were determined using antibodies recognising the HA-epitope tag fused to BTG2. (C) Recruitment of BTG2, but not of BTG2 W103A, to the 3′ UTR of a reporter gene results in reduced mRNA levels. MCF-7 cells were transfected with the indicated expression plasmids. Total RNA was isolated 48 h post transfection and Renilla luciferase mRNA levels determined using RT-qPCR using GAPDH as a reference gene. (D) Recruitment of TOB1, but not of TOB1 W93A, to the 3′ UTR of a reporter gene results in decreased reporter activity. MCF-7 cells were transfected with the indicated expression plasmids. Luciferase activity was determined 48 h post transfection and normalised to total protein content. Protein levels of λN-TOB1 were determined using antibodies recognising the HA-epitope tag fused to TOB1. (E) Recruitment of TOB1, but not of TOB1 W93A, to the 3′ UTR of a reporter gene results in reduced mRNA levels. MCF-7 cells were transfected with the indicated expression plasmids. Total RNA was isolated 48 h post transfection and Renilla luciferase mRNA levels determined using RT-qPCR using GAPDH as a reference gene. * p<0.05. All experiments were carried out in triplicate. Error bars represent the standard error of the mean.

**Figure 6:**
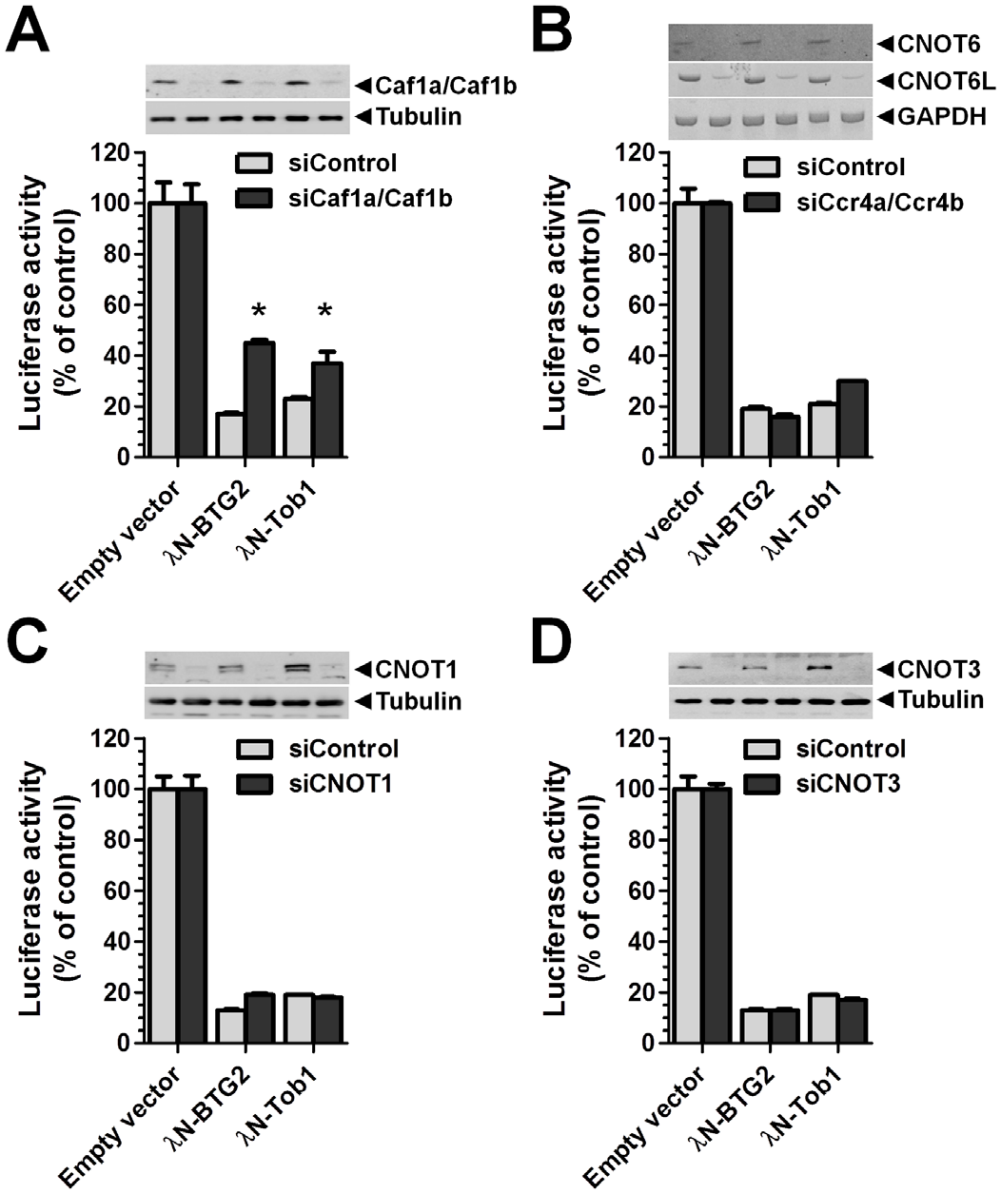
Reduced reporter activity by recruitment of BTG2 or TOB1 is partially rescued by knockdown of the Caf1a/Caf1b deadenylase enzymes. (A) Partial rescue of reporter activity upon combined knockdown of Caf1a/Caf1b. Protein levels were determined using antibodies recognising Caf1a and Caf1b. Tubulin was used as a loading control. (B) Repression of reporter activity upon tethering of λN-BTG2 or λN-TOB1 in combination with combined knockdown of Ccr4a/Ccr4b. Knockdown was confirmed by reverse transcriptase PCR using primers recognising CNOT6 or CNOT6L mRNA. GAPDH was used as a loading control. (C) Repression of reporter activity upon tethering of λN-BTG2 or λN-TOB1 in combination with knockdown of CNOT1. Protein levels were determined using antibodies recognising CNOT1. Tubulin was used as a loading control. (D) Repression of reporter activity upon tethering of λN-BTG2 or λN-TOB1 in combination with knockdown of CNOT3. Protein levels were determined using antibodies recognising CNOT3. Tubulin was used as a loading control. Cells were transfected with siRNAs targeting the indicated components or non-targeting control siRNA. After 48 h, siRNA transfected cell populations were transfected with the reporter mRNA and plasmids expressing λN-BTG2, λN-TOB1, or the empty vector, as indicated. Renilla luciferase activity was determined 24 h after the DNA transfection and normalised against total protein content. * p<0.05. All experiments were carried out in triplicate. Error bars represent the standard error of the mean.

## Results

### BTG2 and TOB1, but not Other BTG/TOB Proteins, Regulate Proliferation of MCF-7 Cells

To identify which of the six BTG/TOB family members (Figure 1A) regulate proliferation of human MCF-7 cells, we first examined the expression of the BTG/TOB genes by mining Affymetrix GeneChip expression data previously obtained [31]. This analysis identified significant levels of BTG1, BTG2, TOB1 and TOB2 mRNAs, and indicated no expression of the BTG3 and BTG4 genes (Figure 1B). This data was confirmed by RT-qPCR analysis (Figure S1). Next, we used siRNA to knockdown the four expressed BTG/TOB members, BTG1, BTG2, TOB1 and TOB2, and assessed the percentage of cells in S-phase as a measure of cell proliferation (Figure 1C, Figure S1). In agreement with the observation that knockdown of TOB1 results in increased proliferation of MCF-7 cells [41], these results showed that knockdown of BTG2 and TOB1, but not BTG1 and TOB2, resulted in increased cell proliferation. It was surprising that this did not correlate with expression level: the mRNA levels of both BTG1 and TOB2 are significantly higher than that of BTG2. To further examine the role of the four expressed BTG/TOB proteins in regulating proliferation of MCF-7 cells, we examined all binary combinations of siRNA-mediated knockdown. Interestingly, combined knockdown of BTG2 and TOB1 resulted in a higher rate of cell proliferation as compared to single knockdown of BTG2 and TOB1 (Figure 1D, p<0.05). No effect on cell proliferation was observed using any other binary combination of BTG/TOB knockdowns (data not shown). We conclude that only BTG2 and TOB1 regulate cell proliferation of MCF-7 cells.

### Identification of Amino Acids of BTG2 and TOB1 Required for the Interaction with the Caf1a and Caf1b Deadenylase Subunits of the Ccr4-Not Complex

To further explore the anti-proliferative activity of BTG2 and TOB1, we aimed to identify amino acids of BTG2 and TOB1 that are required for the interaction with the Caf1a and Caf1b deadenylase subunits of the Ccr4-Not complex. Using sequence alignments of the BTG domain and the crystal structure of the TOB1-Caf1a complex [37], we selected six candidate residues mediating the interactions between BTG2/TOB1 and the deadenylases Caf1a/Caf1b. In addition, we analysed a further amino acid (Trp-103), which was reported during the course of this work as a critical residue for the BTG2-Caf1a interaction [42].

To establish the importance of the selected residues, we generated a series of alanine substitutions and used the yeast two-hybrid assay as a (semi-)quantitative interaction assay. When the interactions between BTG2 and Caf1a were analysed, we confirmed the role of four amino acids (Tyr-65, Asp-75, Trp-103 and Asp-105), which were required for the interaction with Caf1a, and three amino acids (His-53, Glu-108 and Glu-115), whose identity was not important for the binding to Caf1a (Figure 2A). Intriguingly, when the interaction analysis was carried out using Caf1b fused to the activation domain of GAL4, we found that BTG2 H53A did not interact with Caf1b (Figure 2B). To investigate whether the interaction surface of BTG2 was conserved in TOB1, we generated a series of mutants that encode the corresponding set of alanine substitutions. Five substitutions, H45A, F55A, D65A, W93A and D95A, displayed significantly reduced interactions with both Caf1a and Caf1b (Figure 2C and 2D). Consistent with the role of amino acids Glu-108 and Glu-115 of BTG2, the substitution of the corresponding amino acids of TOB1 with alanine, E98A and E105A, did not have a major effect on the interactions with Caf1a and Caf1b (Figure 2C and 2D).

To confirm the role of the identified residues in mediating BTG2-Caf1a interactions in the context of mammalian cells, we also carried out immunoprecipitations. HEK293 cells were transfected with expression plasmids encoding HA-BTG2 and Flag-Caf1a. Following immunoprecipitation using Flag antibodies, small amounts of BTG2 H53A and BTG2 D105A were detected, whereas the levels of BTG2 Y65A, BTG2 D75A and BTG2 W103A were below the limit of detection in anti-Flag immunoprecipitates (Figure 2E). We conclude that from seven candidates, the identity of three residues of BTG2, Tyr-65, Asp-75 and Trp-103, and of their corresponding residues in TOB1, is essential for the interaction with the Caf1a and Caf1b deadenylases. To further explore the functional importance of the interaction between BTG2/TOB1 and Caf1a/Caf1b, we decided to focus on the amino acid substitutions W103A of BTG2 and W93A of TOB1. These amino acid substitutions, which were already partially characterised [42], consistently displayed the most striking effect on the interaction with Caf1a and Caf1b.

### The Anti-proliferative Activity of BTG2 and TOB1 is Mediated via Interactions with the Caf1a and Caf1b Deadenylases

To assess the functional importance of the interactions between BTG2 and the Caf1a/Caf1b deadenylase enzymes, we generated clonal cell lines that express BTG2, or BTG2 W103A, under control of the Tet repressor protein, as well as a clonal cell line transfected with an empty expression cassette. In the presence of doxycycline, similar levels of BTG2 and BTG2 W103A were expressed (Figure 3A). Upon expression of BTG2, cell proliferation was reduced as determined by counting cell numbers. By contrast, no effect on cell proliferation was observed upon expression of BTG2 W103A, or in cells transfected with empty vector (Figure 3B). Moreover, the percentage of cells in S-phase was markedly reduced upon expression of BTG2, which was not detected upon expression of BTG2 W103A, or in cells transfected with empty vector (Figure 3C). In agreement with the notion that expression of BTG2 results in a delayed G1/S transition [43], [44], [45], we confirmed that the reduced fraction of cells in S-phase was accompanied by a concomitant increase in the G1 phase (Table S1).

A reduction in the percentage of cells in S-phase was also observed upon expression of BTG2 following transient transfection, which was not noticed upon expression of BTG2 W103A as compared with empty vector transfected cells (Figure 4A). Consistent with these observations, expression of TOB1, but not of TOB1 W93A, resulted in a reduced percentage of cells in S-phase as compared to empty vector-transfected cells (Figure 4B).

Both BTG2 and TOB1 are phosphorylated at serine residues located outside the BTG domain (Figure 4C). It was reported that the substitution of these amino acids with alanine residues prevents their inactivation and results in increased anti-proliferative activity of these proteins [16], [46]. Therefore, we reasoned that the substitution of the serine residues combined with the alterations W103A of BTG2 or W93A of TOB1 might reveal weak residual anti-proliferative activity of BTG2 and TOB1 that is independent of the interaction with the Caf1a and Caf1b deadenylase enzymes. As expected, expression of BTG2 (S→A) containing the amino acid substitutions S147A and S149A resulted in a reduction of cells in S-phase (compare Figure 4D). By contrast, no effect on S-phase was observed upon expression of BTG2 (S→A) W103A as compared to empty vector-transfected cells (Figure 4D). Similarly, while the percentage of cells in S-phase was reduced upon expression of TOB1 (S→A), no effect on S-phase was observed upon expression of TOB1 (S→A) W93A as compared to cells transfected with empty vector (Figure 4E). In addition, we found that BTG (S→A) and TOB1 (S→A) repress cell proliferation to the same extent as wild type BTG2 and TOB1, respectively (Figure S2). We conclude that phosphorylation does not affect the activity of BTG2 and TOB1 in MCF-7 cells. Taken together, we conclude that the anti-proliferative activity of BTG2 and TOB1 is mediated via interactions with the Caf1a and Caf1b deadenylase enzymes.

### Recruitment of BTG2 or TOB1 to the 3′ UTR of a Reporter mRNA Results in Reduced mRNA Levels and Reporter Activity

To link the role of the BTG/TOB proteins in the regulation of cell proliferation with their function in the regulation of mRNA abundance and translation, we used RNA tethering assays. A Renilla luciferase reporter mRNA containing five BoxB sequences in the 3′ UTR was expressed in combination with a λN peptide, which has high affinity for the BoxB sequences [38], [39], fused to BTG2 or TOB1 (Figure 5A). Recruitment to the 3′ UTR of λN-BTG2 or λN-BTG2 (S→A) resulted in a marked (>80%) reduction of luciferase reporter activity, whereas no reduction of reporter activity was observed upon expression of λN-BTG2 W103A or λN-BTG2 (S→A) W103A (Figure 5B). The level of the mRNA reporter was also reduced upon expression λN-BTG2, albeit to a lesser extent (approximately 60%), which was not observed upon expression of λN-BTG2 W103A (Figure 5C). Similar results were obtained when λN-TOB1 was expressed: λN-TOB1 or λN-TOB1 (S→A) resulted in a marked (>70%) reduction of luciferase reporter activity, whereas no reduction of reporter activity was observed upon expression of λN-TOB1 W103A or λN-TOB1 (S→A) W103A (Figure 5D). Again, the level of the mRNA reporter was also reduced upon expression λN-TOB1, albeit to a lesser extent, which was not observed upon expression of λN-TOB1 W103A (Figure 5E). Together, these results indicate that recruitment of BTG2 or TOB1 to the 3′ UTR results in reduced mRNA translation and mRNA abundance, which is dependent on the interaction with the Caf1a and Caf1b deadenylases.

### Repression of Reporter Activity by Recruitment of BTG2 and TOB1 to the 3′ UTR is Partially Reversed by Knockdown of the Caf1a/Caf1b Deadenylase Enzymes

To find out whether other components of the Ccr4-Not deadenylase contribute to the effects of BTG2 and TOB1 on translation and mRNA abundance, we carried out RNA tethering assays in combination with siRNA-mediated knockdown. Upon combined knockdown of the Caf1a and Caf1b deadenylases, the inhibitory effect of λN-BTG2 and λN-TOB1 on reporter activity was partially reversed, as expected (Figure 6A). However, upon combined knockdown of the Ccr4a and Ccr4b deadenylases, which interact with the Caf1a/Caf1b subunits, no effect on reporter activity was observed (Figure 6B). Similarly, no effect on reporter activity was measured when knockdown was carried out of CNOT1 or CNOT3, two non-catalytic subunits of the Ccr4-Not complex (Figure 6C and 6D). In all cases, knockdown was confirmed by RT-qPCR and/or western blotting (Figure 6 and Figure S3).

Taken together, these results suggest that the activity of BTG2 and TOB1 depends on interactions with the Caf1a and Caf1b deadenylases, and may not be dependent on the activity of the Ccr4a/Ccr4b deadenylases, or on the CNOT1 and CNOT3 non-catalytic subunits of the Ccr4-Not complex.

## Discussion

### The Anti-proliferative Activity of BTG/TOB Proteins is Mediated Through Interactions with the Caf1a and Caf1b Deadenylase Enzymes

There was ambiguity as to whether the anti-proliferative activity of BTG/TOB proteins was related to their ability to interact with the Caf1a and Caf1b deadenylase subunits of the Ccr4-Not complex. Divergent conclusions were obtained using mutations in Caf1a that prevent the interaction of the deadenylase with BTG/TOB proteins [30], [37]. Therefore, we further characterised the interaction surface of BTG2 and TOB1 with the highly related Caf1a and Caf1b enzymes to identify amino acids of BTG/TOB proteins with critical roles in the binding with the deadenylases. One such amino acid of BTG2, Trp-103, was described by Yang et al. during the course of this study [42]. Because the W103A substitution of BTG2, and the homologous amino acid substitution W93A in TOB1, affected the interactions with Caf1a/Caf1b more strongly than any other mutated residue, we focused on this amino acid substitution. Expression of BTG2 or TOB1 in MCF-7 cells inhibited cell proliferation in contrast to BTG2 or TOB1 containing the single amino acid substitution W103A or W93A, respectively. Based on these results, we concluded that the interaction with the Caf1a/Caf1b deadenylases is required for the anti-proliferative activity of BTG2 and TOB1.

This conclusion is entirely consistent with that of Ezzeddine et al., whose results were published during the course of our work [34]. However, Ezzeddine et al used different amino acid substitutions in TOB1 and TOB2 to abolish the interaction with the Caf1a/Caf1b deadenylases: three conserved leucine residues at positions 32, 35 and 36 were changed to glycine [34]. Our work presented here combined with previous reports by Ezzeddine et al. and Horiuchi et al. unambiguously demonstrates that the anti-proliferative activity of BTG/TOB proteins is mediated through interactions with the Caf1a and Caf1b deadenylase enzymes [34], [37].

Previously, we reported that the Caf1a/Caf1b subunits of the Ccr4-Not complex are important for cell proliferation [30]. Thus, Caf1a/Caf1b are not exclusively required for BTG/TOB activity, but also have additional roles. Moreover, it is possible that MCF-7 cells have down-regulated levels of BTG/TOB proteins, a frequently observed phenomenon in breast cancers [11], [41].

### Regulation of BTG/TOB Activity by Phosphorylation

The activity of TOB1 and BTG2 is regulated by phosphorylation of serine residues located in the C-terminal region by the Erk1/Erk2 kinases [16], [46]. Moreover, inactive, phosphorylated TOB1 is frequently found in clinical samples of patients affected by lung and thyroid cancer [8], [9]. In agreement with these observations, we found that expression of BTG2 containing the combined amino acid alterations S147A and S149A appeared to result in a more pronounced decrease in cell proliferation as compared to wild type BTG2. By contrast, alterations of the serine residues that are phosphorylated in TOB1 did not result in increased anti-proliferative activity. In agreement, similar results were obtained by Ezzeddine et al. when the regulation of TOB1 and TOB2 activity by phosphorylation was investigated [34]. We suggest that the inactivation by phosphorylation is redundant with other mechanisms that limit the activity of BTG/TOB proteins in some immortalised cell lines.

### Recruitment of BTG/TOB Proteins to the 3′ UTR Affects Translation and mRNA Abundance

The recruitment of TOB1 to the 3′ UTR of mRNA involves interactions with RNA-binding proteins, which results in reduced mRNA stability [35], [36]. Moreover, artificial recruitment of TOB1 and TOB2 promotes deadenylation by binding the Ccr4-Not deadenylase [34]. Our observations that tethering of BTG2 or TOB1 to the 3′ UTR results in decreased translation and mRNA abundance are consistent with a role for BTG2 and TOB1 as a recruitment factor of the Ccr4-Not deadenylase [34]. However, it is presently unclear how BTG2 may bind to the 3′ UTR region of mRNAs. Whereas TOB1 and TOB2 have large C-terminal regions that mediate interactions with poly(A)-binding proteins and CPEB3, BTG2 and the closely related BTG1 protein only have a short C-terminal region [3], [35], [36].

It was unclear which catalytic subunit contributes to deadenylation of mRNA upon recruitment by BTG/TOB proteins. Both the DEDD/Caf1 and EEP/Ccr4-type subunits are active deadenylase enzymes in vitro. However, despite the fact that these proteins directly interact with each other, the abundance of different sets of mRNAs is regulated by each type [30], [31]. While the use of BTG/TOB mutants identified the requirement for interactions with the Caf1a/Caf1b subunit in promoting deadenylation, the active enzymatic subunit, or the role of other non-catalytic subunits, was not assessed. To this end, we combined siRNA knockdown with RNA tethering assays. Whereas combined knockdown of Caf1a/Caf1b partially rescued the decrease of reporter activity upon recruitment of BTG2 or TOB1, no effect was observed following knockdown of the Ccr4a/Ccr4b deadenylase subunits, or upon knockdown of the non-catalytic subunits CNOT1, which acts as a central platform and is the only essential subunit in yeast, or CNOT3. One possibility, however, that cannot be excluded at this stage, is whether the BTG/Tob proteins sequester the Caf1-type subunits in the absence of other components of the Ccr4-Not complex rather than recruitment of the Ccr4-Not complex through Caf1a/Caf1b.

### Unique or Overlapping Roles of the BTG/TOB Proteins in the Regulation of Cell Proliferation?

In MCF-7 cells, four out of six BTG/TOB genes are expressed. Surprisingly, the anti-proliferative activity did not correlate with mRNA levels: BTG2 was expressed at significantly lower levels than TOB1, but BTG2 knockdown increased proliferation to a similar extent as knockdown of TOB1. Furthermore, both BTG1 and TOB2 were expressed at higher levels than BTG2, but knockdown of either did not affect cell proliferation. Interestingly, combined knockdown of BTG2 and TOB1 resulted in further enhanced proliferation. It will be of interest to establish whether BTG2 and TOB1 regulate the abundance and translation of a similar group of mRNAs and influence cell proliferation through similar mechanisms, or whether BTG2 and TOB1 regulate the abundance and translational efficiency of distinct mRNA sets and regulate cell cycle progression by different mechanisms.

## Supporting Information

Figure S1 **(A) Confirmation of BTG/TOB expression by reverse transcriptase quantitative PCR.** (B) Relative mRNA levels of BTG1, BTG2, TOB1 and TOB2 upon siRNA-mediated knockdown in MCF7 cells.
(TIF)

Figure S2 **Phosphorylation of BTG2 and TOB1 does not affect their activity in MCF-7 cells.** (A) Expression of wild type BTG2 and BTG2 (S→A) inhibits proliferation of MCF-7 cells to a similar extent. (B) Expression of wild type TOB1 and TOB1 (S→A) inhibits proliferation of MCF-7 cells to a similar extent. The p-values comparing wild type and (S→A) mutants are shown.
(TIF)

Figure S3 **Analysis of knockdown efficiency upon treatment with (A) combined Caf1a/Caf1b siRNA; (B) combined Ccr4a/Ccr4b siRNA; (C) CNOT1 siRNA; (D) CNOT3 siRNA by reverse transcriptase quantitative PCR.** Total mRNA was isolated 48 h after siRNA transfection. Levels of the indicated mRNAs were determined using GAPDH as a reference.
(TIF)

Table S1 **Distribution of cell cycle phases in MCF-7 cells expressing BTG2, BTG2 W103A, or transfected with empty vector.**
(DOC)
