# Supplementary figures and images for: The Anti-Proliferative Activity of BTG/TOB Proteins Is Mediated via the Caf1a (CNOT7) and Caf1b (CNOT8) Deadenylase Subunits of the Ccr4-Not Complex
Source: PLoS One. 2012 Dec 7;7(12):e51331. doi: 10.1371/journal.pone.0051331 (PMC3517456; doi:10.1371/journal.pone.0051331)

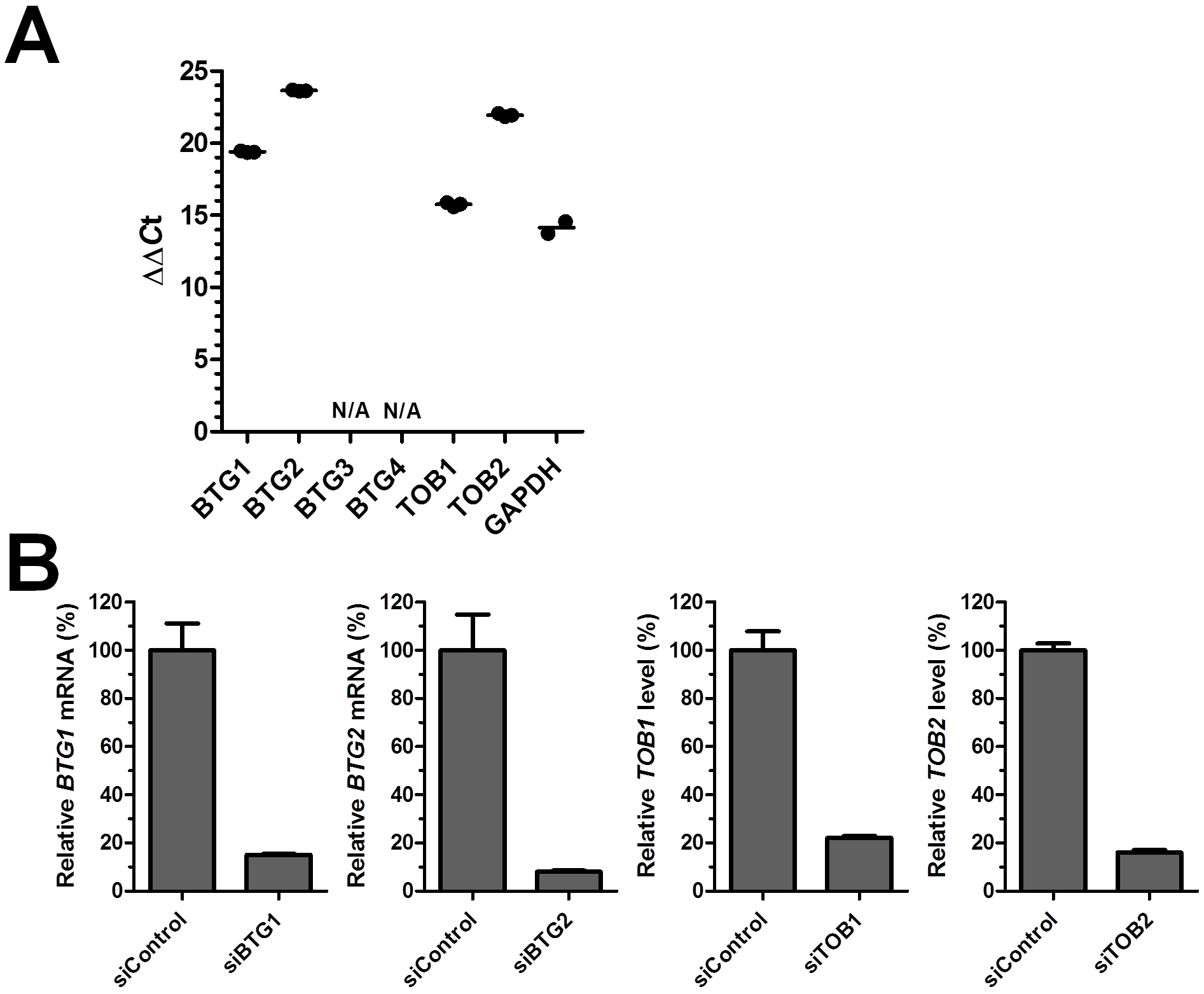

Supplement: Figure S1 — (A) Confirmation of BTG/TOB expression by reverse transcriptase quantitative PCR. (B) Relative mRNA levels of BTG1, BTG2, TOB1 and TOB2 upon siRNA-mediated knockdown in MCF7 cells. (TIF) [file pone.0051331.s001.tif]

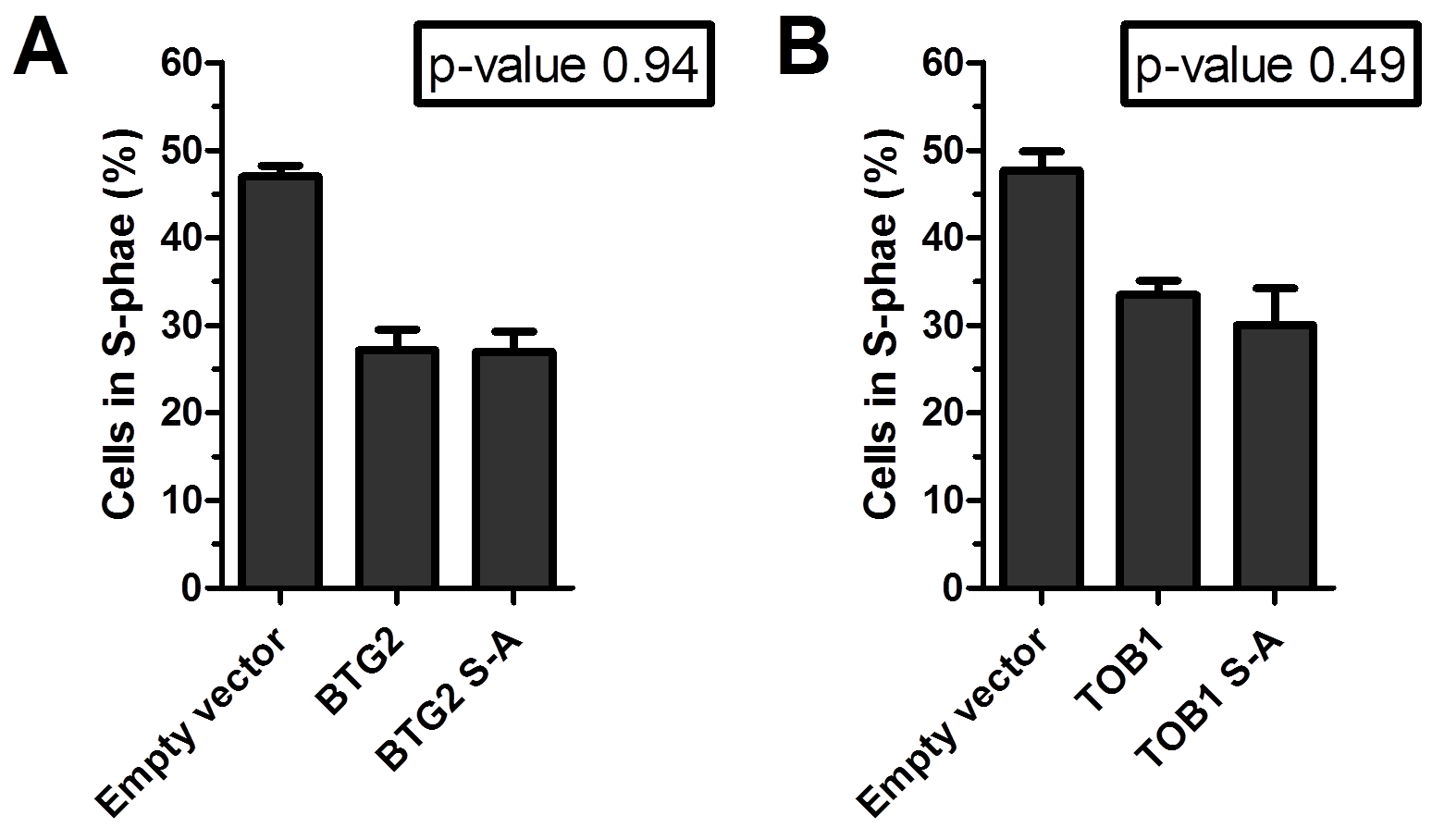

Supplement: Figure S2 — Phosphorylation of BTG2 and TOB1 does not affect their activity in MCF-7 cells. (A) Expression of wild type BTG2 and BTG2 (S→A) inhibits proliferation of MCF-7 cells to a similar extent. (B) Expression of wild type TOB1 and TOB1 (S→A) inhibits proliferation of MCF-7 cells to a similar extent. The p-values comparing wild type and (S→A) mutants are shown. (TIF) [file pone.0051331.s002.tif]

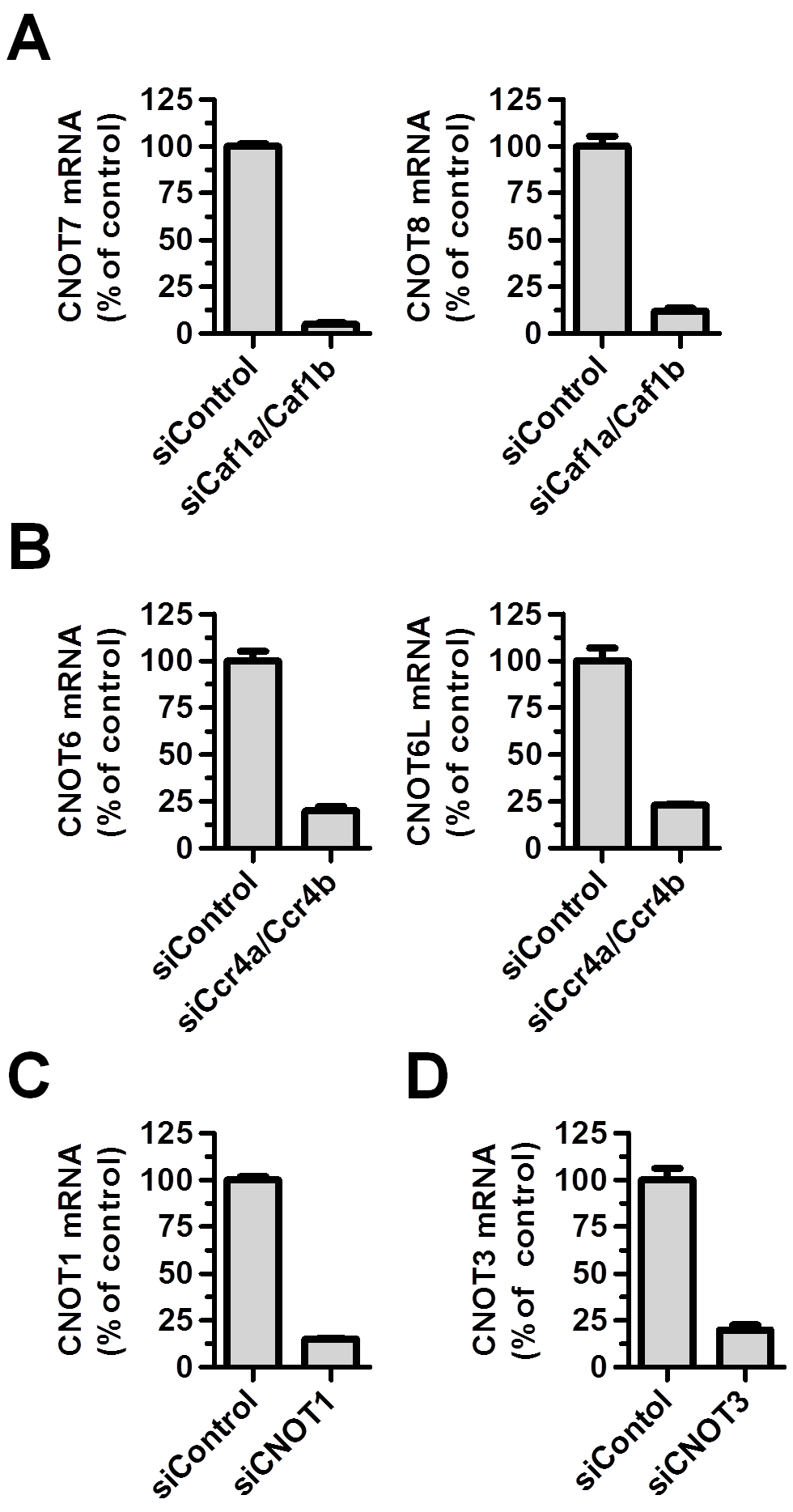

Supplement: Figure S3 — Analysis of knockdown efficiency upon treatment with (A) combined Caf1a/Caf1b siRNA; (B) combined Ccr4a/Ccr4b siRNA; (C) CNOT1 siRNA; (D) CNOT3 siRNA by reverse transcriptase quantitative PCR. Total mRNA was isolated 48 h after siRNA transfection. Levels of the indicated mRNAs were determined using GAPDH as a reference. (TIF) [file pone.0051331.s003.tif]
