# Supplementary material for: The Anti-Proliferative Activity of BTG/TOB Proteins Is Mediated via the Caf1a (CNOT7) and Caf1b (CNOT8) Deadenylase Subunits of the Ccr4-Not Complex
Source: PLoS One. 2012 Dec 7;7(12):e51331. doi: 10.1371/journal.pone.0051331 (PMC3517456; doi:10.1371/journal.pone.0051331)
Supplement: Table S1 — Distribution of cell cycle phases in MCF-7 cells expressing BTG2, BTG2 W103A, or transfected with empty vector. (DOC) [file pone.0051331.s004.doc]

**Supporting Information, Table S1**

|  | **Empty vector** | **BTG2** | **BTG2 W103A** |
| --- | --- | --- | --- |
| **G_1_** | 67.5 ± 0.14 | *73.25 ± 1.63^*^* | 62.6 ± 3.96 |
| **S** | 28.4 ± 0.71 | *22.35 ± 1.06^*^* | 33.35 ± 2.90 |
| **G_2_/M** | 4.1 ± 0.85 | 4.45 ± 0.66^*^ | 4.05 ± 1.06 |

Distribution of cell cycle phases in MCF-7 cells expressing BTG2, BTG2 W103A, or transfected with empty vector (n=2). *^*^ p<0.05* as compared to cells transfected with empty vector.
